# Supplementary figures and images for: Compound Biejia-Ruangan tablets activate the STING-TBK1 pathway to alleviate hepatic fibrosis in alveolar echinococcosis
Source: Microbiol Spectr. 2026 Apr 21;14(6):e02115-25. doi: 10.1128/spectrum.02115-25 (PMC13228014; doi:10.1128/spectrum.02115-25)

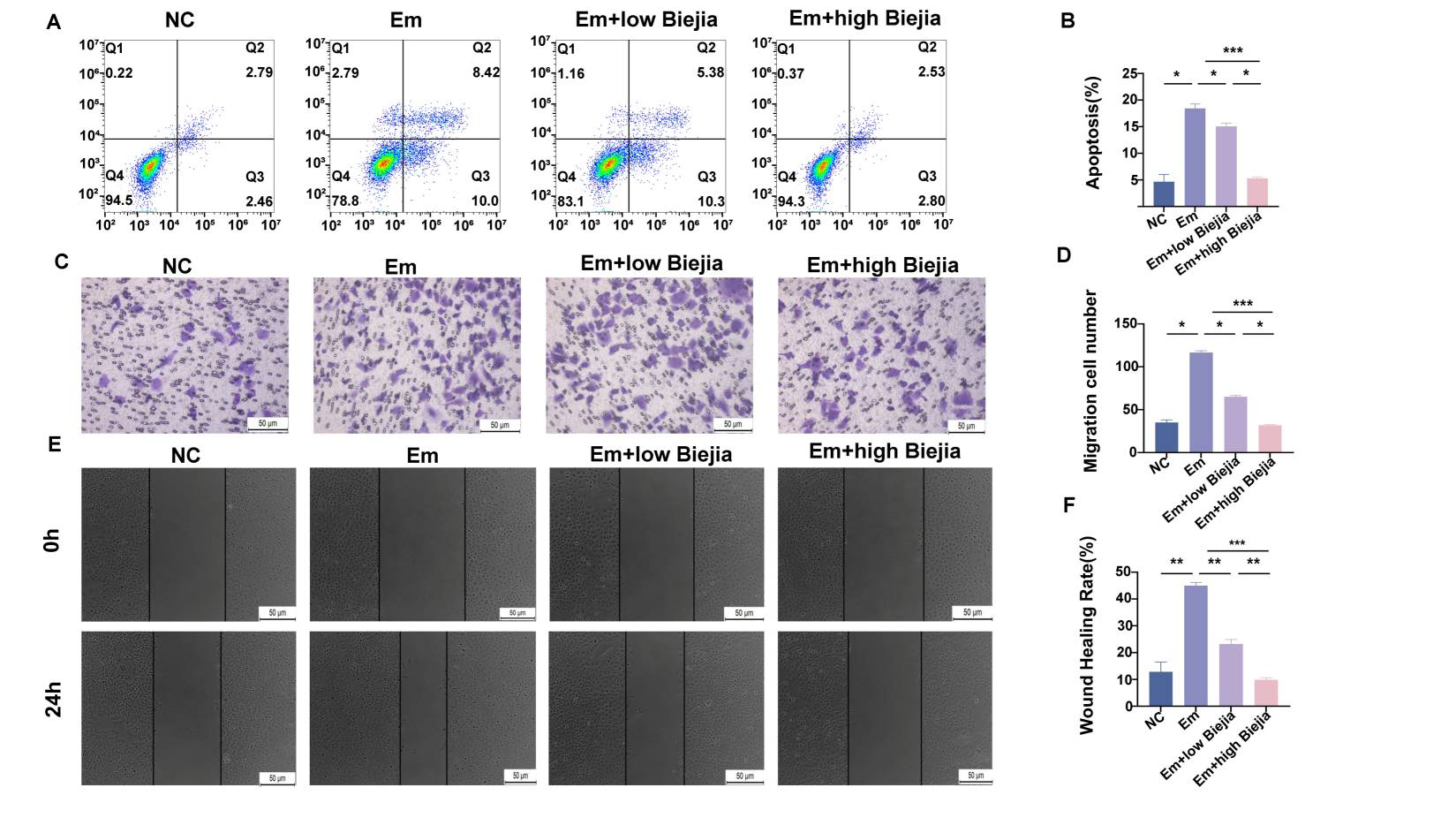

Supplement: Fig. S1 — Effects of CBRT-containing serum on apoptosis of GHA1 cells and migration of RAW264.7 macrophages in an in vitro E.m. mode. [file spectrum.02115-25-s0001.tif]

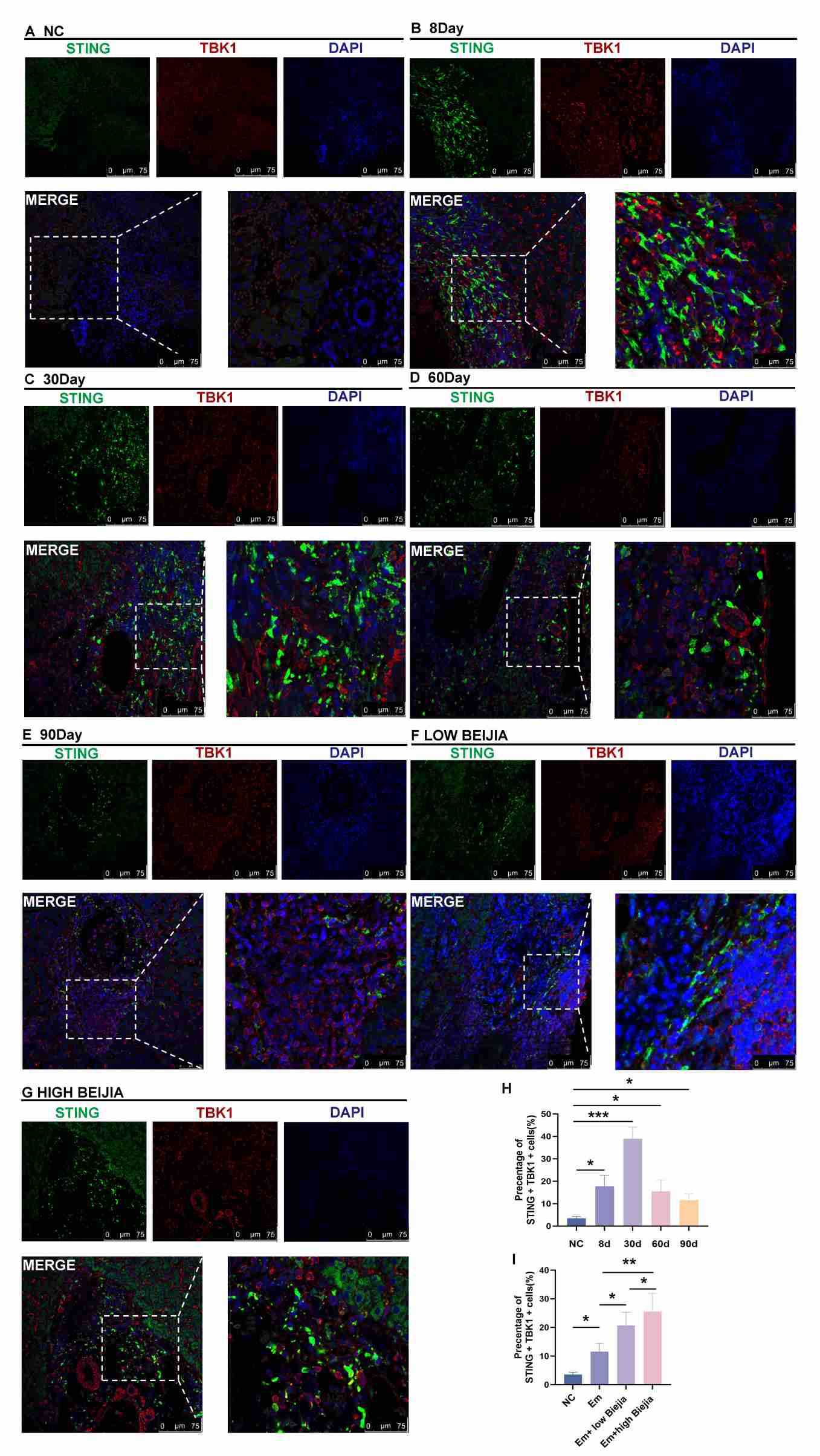

Supplement: Fig. S2 — Dynamic co-localization of STING and TBK1 in hepatic tissues during E.m. progression. [file spectrum.02115-25-s0002.tif]
