# Supplementary material for: Compound Biejia-Ruangan tablets activate the STING-TBK1 pathway to alleviate hepatic fibrosis in alveolar echinococcosis
Source: Microbiol Spectr. 2026 Apr 21;14(6):e02115-25. doi: 10.1128/spectrum.02115-25 (PMC13228014; doi:10.1128/spectrum.02115-25)
Supplement: Supplemental material — Supplemental figure legends. [file spectrum.02115-25-s0003.docx]

**Supplement Figure legends**

**Fig.S1.** **Effects of CBRT-containing serum on apoptosis of GHA1 cells and migration of RAW264.7 macrophages in an in vitro *E.m.* model. A-B.** Analysis of apoptosis in GHA1 liver cells. A, Representative flow cytometry plots showing apoptotic cell populations in each treatment group. B, Quantitative analysis of total apoptosis rates. **C-F.** Analysis of migration and invasion capacities of RAW264.7 macrophages. C, D, Representative images from Transwell migration (C) and invasion (D) assays. E, F, Corresponding quantitative analysis of migrated (E) and invaded (F) cell counts. Data are from three biologically independent replicates (n = 3) and presented as mean ± SEM. Statistical significance was determined using unpaired Student’s t-test (for comparisons between two groups) or one-way ANOVA (for multi-group comparisons followed by appropriate post-hoc tests), as indicated: **P* < 0.05, ***P* < 0.01, ****P* < 0.001.

**Fig.S2. Dynamic co-localization of STING and TBK1 in hepatic tissues during *E.m.* progression**

A-G. Analysis of STING and TBK1 co-localization by immunofluorescence. N, Representative immunofluorescence images showing co-localization of STING (green) and TBK1 (red) in liver tissues at different time points; nuclei are counterstained with DAPI (blue). H-I, Quantitative analysis of STING and TBK1 co-localization. Data are from three biologically independent replicates (n = 3) and presented as mean ± SEM. Statistical significance was determined using unpaired Student’s t-test (for comparisons between two groups) or one-way ANOVA (for multi-group comparisons followed by appropriate post-hoc tests), as indicated: **P* < 0.05, ***P* < 0.01, ****P* < 0.001.
