# Supplementary material for: Compound Biejia-Ruangan tablets activate the STING-TBK1 pathway to alleviate hepatic fibrosis in alveolar echinococcosis
Source: Microbiol Spectr. 2026 Apr 21;14(6):e02115-25. doi: 10.1128/spectrum.02115-25 (PMC13228014; doi:10.1128/spectrum.02115-25)
Supplement: Table S1 — Manufacturer and article number of reagents used in research. [file spectrum.02115-25-s0004.docx]

**Supporting Information Antibodies for immunohistochemistry (IHC)**

| **Antibody** | **Source** | **Catalog Number** | **Concentration** |
| --- | --- | --- | --- |
| α-SMA | Bioss | Bsm-33188M | 1:200 |
| Collagen Ⅰ | Affinity | AF7001 | 1:100 |
| INOS | Bioss | bs-0086R | 1:200 |
| Arg-1 | proteintech | 16001-1-AP | 1:5000 |
| STING | Wanleibio | WL03663 | 1:200 |
| TBK1 | Wanleibio | WLH3384 | 1:200 |

**Supporting Information Antibodies for Western blot (WB)**

| **Antibody** | **Source** | **Catalog Number** | **Concentration** |
| --- | --- | --- | --- |
| α-SMA | Bioss | Bsm-33188M | 1:1000 |
| Collagen I | Bioss | bs-0086R | 1:1000 |
| Arg-1 | proteintech | 16001-1-AP | 1:5000 |
| STING | Wanleibio | WL03663 | 1:500 |
| TBK1 | Wanleibio | WL02966 | 1:500 |
| INOS | Abcam | ab191606 | 1:1000 |
| GAPDH | proteintech | 25128-1-AP | 1:1000 |

**Supporting Information Antibodies for ELISA**

**（NanjingLapuda Biotechnology Co., Ltd）**

| **Antibody** | **Source** | **Catalog Number** |
| --- | --- | --- |
| IL-2 | LAPUDA | LA128808H |
| IL-4 | LAPUDA | LA128809H |
| IL-10 | LAPUDA | LA128803H |
| IFN-γ | LAPUDA | LA128805H |

**Supporting Information Primer sequences for qRT-PCR**

Primer sequence of human

| Gene（human） | （5’→3’） |
| --- | --- |
| hα-SMA | Forward: CCCAGACATCAGGGAGTAATGG |
|  | Reverse: TCTATCGGATACTTCAGCGTCA |
| hINOS | Forward: GGCCAGATCCTGTCCAAGC |
|  | Reverse: GTGGGTTTCCACCATTAGCAC |
| hArg-1 | Forward: GTGGAAACTTGCATGGACAAC |
|  | Reverse: AATCCTGGCACATCGGGAATC |
| hSTING | Forward: CAGCCAGATGCAATCAATGCC |
|  | Reverse: TGGAATCCTGAACCCACTTCT |
| hTBK1 | Forward: CCACATCTCGTTCTCGGTTTATC |
|  | Reverse: CAGGGAGCACCGTAATCATAATC |
| hCollahen I | Forward: TATTTGGACTTTGCGACAAGACT |
|  | Reverse: TCGAACGTACTGGTCTGGATAG |
| hGAPDH | Forward: GCACCGTCAAGGCTGAGAAC |
|  | Reverse: TGGTGAAGACGCCAGTGGA |

Primer sequence of mouse

| Gene（mouse） | （5’→3’） |
| --- | --- |
| mα-SMA | Forward: TTCGTGACTACTGCCGAGC |
|  | Reverse: GTCAGGCAGTTCGTAGCTCT |
| mINOS | Forward: GTGTGGAGCAACATGTGGAACTCTA |
|  | Reverse: TTGGTTCAGCCACTGCCGTA |
| mArg-1 | Forward: CTCCAAGCCAAAGTCCTTAGAG |
|  | Reverse: GGAGCTGTCATTAGGGACATCA |
| mSTING | Forward: TTAAAAACCTGGATCGGAACCAA |
|  | Reverse: GCATTAGCTTCAGATTTACGGGT |
| mTBK1 | Forward: ATCCACGGCATACTATCAACATC |
|  | Reverse: CTGCCTGCGACAGATGAGTG |
| mCollagen I | Forward: TCCGATTACCAAGTGCTCTTTC |
|  | Reverse: GCAGCAAAGGCTTCTGGGATAA |
| mGAPDH | Forward: AGGTCGGTGTGAACGGATTTG |
|  | Reverse: GGGGTCGTTGATGGCAACA |
